# The different association between fat mass distribution and intake of three major nutrients in pre- and postmenopausal women

**DOI:** 10.1371/journal.pone.0304098

**Published:** 2024-05-29

**Authors:** Qin Sun, Congrong Liu, Shuxin Li, Juanjuan Ren, Zhaoxia Wang

**Affiliations:** Department of Obstetrics and Gynecology, Yulin Hospital, The First Affiliated Hospital of Xi’an Jiaotong University, Xi’an, China; Shahjalal University of Science and Technology, BANGLADESH

## Abstract

**Background:**

Obesity, characterized by excessive body fat accumulation, is associated with various chronic health conditions. Body fat plays a crucial role in health outcomes, and nutrient intake is a contributing factor. Menopause further influences body fat, but the precise relationships between nutrients and fat mass distribution in pre- and post-menopausal women are unclear.

**Methods:**

Data from 4751 adult women aged ≥18 years old (3855 pre-menopausal, 896 post-menopausal) with completed information were obtained from the National Health and Examination Survey (NHANES) from 2011 to 2018. Multivariate linear regression models were used to examine the associations between protein, carbohydrate, fat intake and total percent fat (TPF), android percent fat (APF), gynoid percent fat (GPF), android to gynoid ratio (A/G), subcutaneous adipose tissue mass (SAT), visceral adipose tissue mass (VAT). Subgroup analyses, stratified by menopausal status, were also conducted. Additionally, we employed smoothing curve fitting techniques to investigate potential non-linear relationships between fat mass distribution and nutrient intake.

**Results:**

Compared with pre-menopausal women, post-menopausal women had higher body fat, BMI, and metabolic indicators but lower nutrient intake (All p<0.05). In the overall analysis, we found significant correlations between nutrient intake and fat mass. Specifically, protein intake was negatively correlated with TPF (β = -0.017, 95% CI: -0.030, -0.005), APF (β = -0.028, 95% CI: -0.044, -0.012), GPF (β = -0.019, 95% CI: -0.030, -0.008), while fat intake showed positive correlations with these measures (SAT: β = 2.769, 95% CI: 0.860, 4.678). Carbohydrate intake exhibited mixed associations. Notably, body fat mass-nutrient intake correlations differed by menopausal status. Generally speaking, protein intake showed negative correlations with body fat distribution in pre-menopausal women but positive correlations in post-menopausal women. Carbohydrate intake revealed significant negative associations with abdominal and visceral fat in post-menopausal women, while fat intake was consistently positive across all fat distribution indices, especially impacting visceral fat in post-menopausal women.

**Conclusion:**

Dietary intake plays a crucial role in body fat distribution, with menopausal status significantly influencing the impact of nutrients on specific fat distribution metrics. The study emphasizes the need for dietary guidelines to consider the nutritional needs and health challenges unique to women at different life stages, particularly concerning menopausal status, to effectively manage obesity.

## Introduction

Obesity, characterized by excessive body fat accumulation, is strongly correlated with various chronic health conditions such as diabetes, dyslipidemia, hypertension and cardiovascular disease [1–3]. The global prevalence of obesity has nearly tripled since 1975, and it is projected that one in five adults will be obese by 2025 [4].

Body mass index (BMI), commonly utilized as an obesity index with a threshold of 30 kg/m^2^, has limitations in predicting the risk of metabolic diseases as emerging evidence suggests that fat distribution, rather than just fat mass, is more strongly associated with these conditions [5]. To address this limitation, the application of dual-energy x-ray absorptiometry (DXA) has gained significant attention among researchers. First introduced by Wilson in 2012, DXA represents an accurate approach for estimating body mass and volume, enabling the precise quantification of adiposity distribution [6].

Furthermore, the impact of energy intake and dietary sources on obesity has been widely recognized [7, 8]. Notably, the roles of the three major nutrients differ in this regard. Protein intake has been shown to contribute to the preservation of muscle mass [9], and diets rich in healthy carbohydrates high in fiber may have a beneficial effect on muscle health [10]. Conversely, high dietary fat intake has been associated with increased energy intake, body weight, and is considered a significant contributor to obesity pathogenesis [11]. Given these multifaceted factors, understanding the complex interplay between fat mass distribution, nutrient intake, and their respective impacts on health outcomes is crucial for devising effective preventive and therapeutic strategies.

Menopause, characterized by the decline in estrogen levels, exerts a significant effect on body fat mass in postmenopausal women, with studies indicating an increase in fat mass among aging women [12, 13]. However, despite the growing body of research in this field, the precise relationships between the three major nutrients and body fat distribution in both pre- and post-menopausal adult women remain uncertain. Previous studies have investigated aspects of nutrient intake and body composition, yet limitations exist, including small sample sizes, inconsistent methodologies, and limited representation of diverse populations [14–16]. Many studies have primarily focused on the relationship between dietary factors and overall obesity without delving into the nuanced aspects of fat mass distribution and its implications for health outcomes [17, 18].

Recognizing the gaps in our current understanding, our study was designed to comprehensively examine the associations between body fat mass and its distribution with protein, carbohydrate, and fat intake in women from a large and diverse US general population based on data from the National Health and Nutrition Examination Survey (NHANES). Furthermore, we conducted subgroup analyses based on menopausal status to explore how dietary intake and fat distribution differ between premenopausal and postmenopausal women. Our study aims to test the following hypotheses:

1. We hypothesize that there will be significant associations between protein, carbohydrate, and fat intake and various measures of body fat distribution.
2. We hypothesize that these associations will vary between premenopausal and postmenopausal women, with potential differences in the impact of nutrient intake on fat distribution.
3. We hypothesize that our findings will provide valuable insights into the development of evidence-based dietary guidelines tailored specifically for women at different stages of their reproductive life, with a focus on addressing their unique nutritional needs and health challenges.

By testing these hypotheses, our study seeks to contribute to a better understanding of the intricate interplay between diet, body composition, and health outcomes in women, ultimately aiming to inform strategies for obesity prevention and management.

## Materials and methods

### Study population

**Fig 1:**
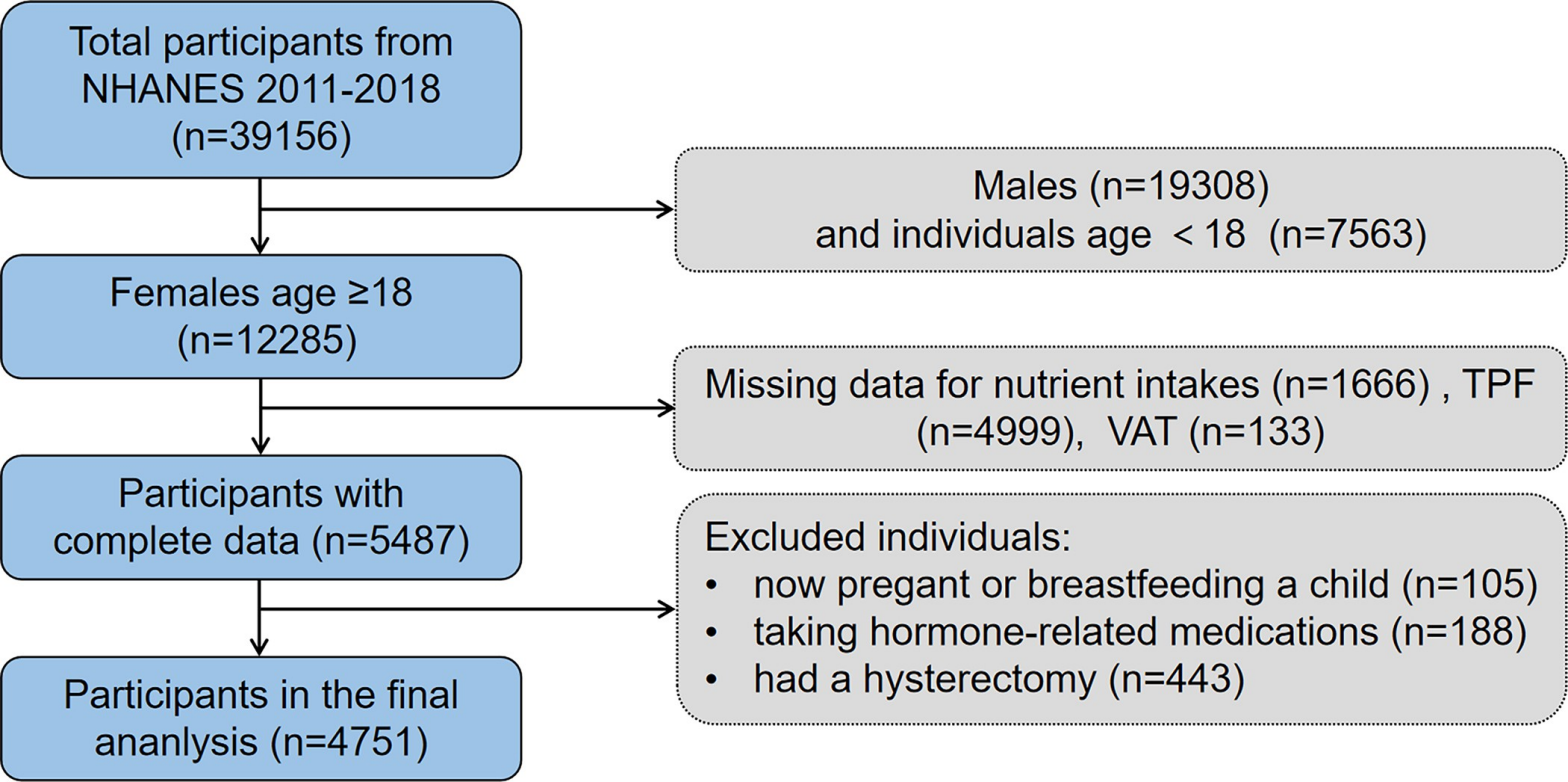
Flow chat of sample selection from the NHANES.

A total of 39156 participants from NHANES conducted between 2011 and 2018 were initially included in our study. Among them, 12285 female participants aged 18 years and older were identified for further analysis. To ensure data integrity, participants with incomplete information on nutrient intake (n = 1666) and fat mass (n = 5132) were excluded. An additional 736 participants were excluded due to self-reported pregnancy or breastfeeding a child (n = 105) or the sue of hormone-related medications (n = 188) or had a hysterectomy (n = 443). Finally, 4751 subjects were enrolled in this analysis. A schematic representation of the sample selection process is depicted in **Fig 1.**

We adhered to the principles outlined in the NHANES study design and data collection. The NHANES study obtained ethical clearance from the Research Ethics Review Board of the National Center for Health Statistics (Protocol #2011–17). Further information can be found at https://www.cdc.gov/nchs/nhanes/irba98.htm.

### Variables

#### Outcomes

The outcome variables in our study encompassed total body fat and its distribution: total percent fat (TPF), android percent fat (APF), gynoid percent fat (GPF), android to gynoid ratio (A/G), subcutaneous adipose tissue mass (SAT), and visceral adipose tissue mass (VAT) [19–21]. These measurements were conducted using the Hologic Discovery model A densitometers (Hologic, Inc., Bedford, Massachusetts) during the 2011–2018 NHANES survey, using software version Apex 3.2. The radiation exposure from DXA whole body scans is extremely low at less than 20 uSv. All scans were analyzed with Hologic APEX version 4.0 software with NHANES BCA option. The DXA examinations were administered by trained and certified radiology technologists. Further details of the DXA examination protocol are documented in the Body Composition Procedures Manual located on the NHANES website. (https://wwwn.cdc.gov/nchs/nhanes/ContinuousNhanes/Manuals.aspx?BeginYear=2017).

**Fig 2:**
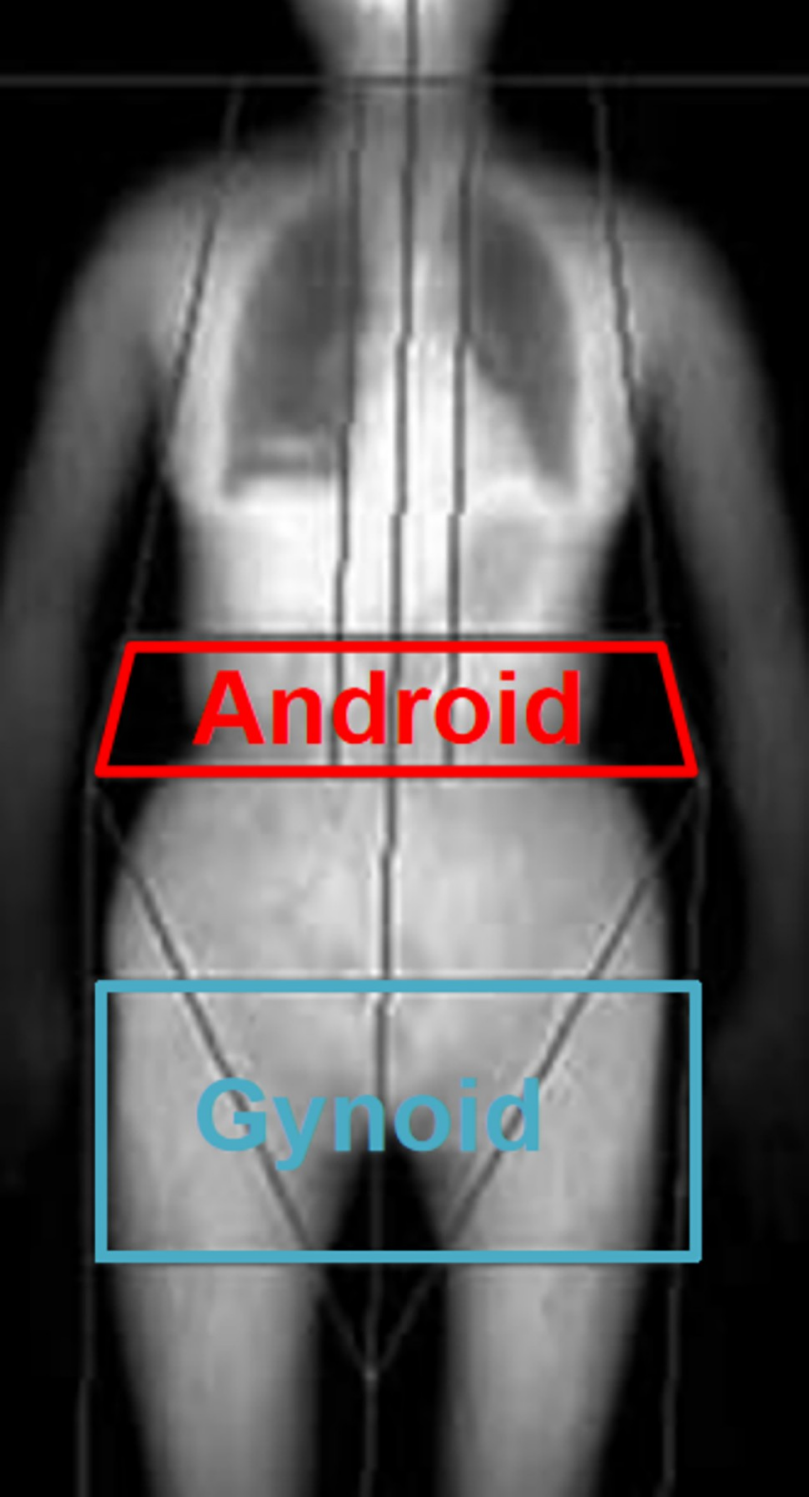
Regions of android fat, and gynoid fat assessed by DXA [49].

TPF was calculated as the ratio of total fat mass to the combined mass of fat and lean tissue. APF and GPF were the ratios of android fat mass to android total mass and gynoid fat mass to gynoid total mass, respectively and A/G ratio was determined by dividing the android fat mass by the gynoid fat mass. For the purpose of anatomical definition, the android area was delineated as the lower trunk region enclosed by two lines: the pelvic horizontal cut line at its lower boundary and an automatically placed line positioned above the pelvic line. The upper gynoid line was positioned 1.5 times the height of the android region below the pelvic line, while the lower gynoid line was positioned to maintain a distance between the two gynoid lines equivalent to twice the height of the android region (The detailed DXA partition diagram is showed in the Fig 2). Measurements of VAT mass, representing the fat within the abdominal cavity, were taken at the approximate interspace location between the L4 and L5 vertebrae. Similarly, measurements of SAT mass, indicative of fat located outside the abdominal cavity, were obtained at the approximate interspace location between the L4 and L5 vertebrae [22, 23].

#### Exposures

The exposure variables in our study comprised the intake of three major nutrients: protein, carbohydrates, and fats. In the 2011–2018 NHANES survey, all participants are eligible for two 24-hour dietary recall interviews. The first dietary recall interview is collected in-person in the Mobile Examination Center (MEC) and the second interview is collected by telephone 3 to 10 days later. During these interviews, participants were asked to provide a comprehensive record of all foods and beverages consumed over the previous 24-hour period, spanning from midnight to midnight. Nutrient estimations were calculated using cycle-specific versions of the US Department of Agriculture (USDA) Food and Nutrition Database for Dietary Studies, ensuring the accuracy and reliability of the nutrient intake data [24, 25]. The examination protocol and data collection methods are fully documented in the NHANES dietary interviewer procedures manuals.

#### The definition of menopause

Menopause, characterized by the diminished secretion of ovarian hormones estrogen and progesterone, is clinically diagnosed after 12 consecutive months of amenorrhea unrelated to any underlying pathology. The average age at which women experience their final menstrual period in the United States is reported to be around 50–51 years [26]. Therefore, for the purposes of our study and based on a review of the literature, menopause was retrospectively defined as the cessation of natural menstrual cycles for a minimum of 12 months in women aged 50 years and older [27–30].

#### Other variables

Total energy intake was calculated as the cumulative sum of energy derived from protein, carbohydrates, and fat. Additional variables encompassed sociodemographic factors (age, race), physical examinations (BMI, arm circumference, waist circumference), parity (how many times you have been pregnant), health-related behaviors (smoking status, heavy alcohol, vigorous work activity), and the presence of certain medical conditions (hypertension, diabetes, hypercholesterolemia). Furthermore, several metabolic indicators were considered, including HDL cholesterol, LDL cholesterol, total cholesterol, triglycerides, creatinine, blood urea nitrogen, serum uric acid, serum glucose and glycohemoglobin.

#### Statistical analyses

The baseline characteristics of the participants were presented as mean values ± standard deviation for continuous variables and as percentages for categorical variables, stratified by menopausal status. To investigate the relationship between fat mass distributions and the intake of three major nutrients, multivariable regression models were employed. Adjustments were made for variables including age, race, energy intake, parity, hypertension, hypercholesterolemia, diabetes, smoking status, heavy alcohol and vigorous work activity. Subgroup analyses were conducted by performing multivariable regression models stratified by menopausal status. Additionally, to address potential non-linear relationships between body fat and nutrient intake, smooth curve fitting techniques were applied [31–33]. All statistical analyses were carried out using R (http://www.Rproject.org) and EmpowerStats (http://www.empowerstats.com).

## Results

### Participant characteristics

In the final analysis, a total of 4751 women aged 18 years or older were included. Of these, 3855 (mean age: 32.40 ± 9.59) were pre-menopausal and 896 (mean age: 54.08 ± 2.83) were post-menopausal women. Compared to the pre-menopausal group, the post-menopausal group exhibited higher levels of body fat (TPF, APF, GPF, A/G, SAT, and VAT), BMI, arm circumference, and waist circumference. They also had higher levels of metabolic indicators (HDL, LDL, TC, TG, creatinine, blood urea nitrogen, serum uric acid, serum glucose and glycohemoglobin). In terms of dietary intake, post-menopausal women had lower intakes of energy and nutrients (protein, carbohydrate, and fat). Furthermore, a higher proportion of postmenopausal women were observed to be smokers, suffer from hypertension, diabetes, hypercholesterolemia, and be pregnant. Conversely, fewer postmenopausal women engaged in vigorous physical activity **(Table 1).**

**Table 1:** Baseline characteristics of 4751 participants according to menopausal status.

Characteristics	Menopause		P value
	No (n = 3855)	Yes (n = 896)	
Total percent fat (%)	37.89 ± 6.43	40.22 ± 5.50	<0.001
Android percent fat (%)	37.84 ± 8.51	40.07 ± 7.34	<0.001
Gynoid percent fat (%)	41.82 ± 5.33	42.86 ± 5.05	<0.001
Android to gynoid ratio	0.90 ± 0.15	0.94 ± 0.15	<0.001
Subcutaneous adipose tissue (g)	1880.75 ± 827.25	1943.64 ± 717.04	0.04
Visceral adipose tissue (g)	398.50 ± 249.74	589.37 ± 294.51	<0.001
Protein intake (g)	71.19 ± 29.33	68.58 ± 26.60	0.01
Carbohydrate intake (g)	225.84 ± 91.46	214.40 ± 82.93	<0.001
Fat intake (g)	71.51 ± 33.85	67.81 ± 31.16	<0.01
Energy intake (kcal)	1847.97 ± 691.11	1747.83 ± 608.03	<0.001
Age (years)	32.40 ± 9.59	54.08 ± 2.83	<0.001
BMI (kg/m^2^)	28.52 ± 7.51	29.58 ± 7.21	<0.001
Arm circumference (cm)	31.81 ± 5.56	32.33 ± 5.25	0.01
Waist circumference (cm)	93.21 ± 17.11	97.75 ± 15.85	<0.001
HDL Cholesterol (mmol/L)	1.44 ± 0.38	1.54 ± 0.46	<0.001
LDL Cholesterol (mmol/L)	2.70 ± 0.78	3.25 ± 0.97	<0.001
Triglycerides (mmol/L)	1.33 ± 1.46	1.69 ± 1.30	<0.001
Total Cholesterol (mmol/L)	4.67 ± 0.93	5.42 ± 1.09	<0.001
Creatinine (μmol/L)	63.47 ± 19.61	66.68 ± 26.09	<0.001
Blood urea nitrogen (mmol/L)	3.91 ± 1.37	4.74 ± 1.65	<0.001
Serum uric acid (μmol/L)	269.21 ± 63.39	289.42 ± 71.56	<0.001
Serum glucose (mmol/L)	5.18 ± 1.52	5.97 ± 2.97	<0.001
Glycohemoglobin (%)	5.43 ± 0.84	6.01 ± 1.40	<0.001
Race (%)			0.609
Hispanic	1045 (27.11%)	257 (28.68%)	
Non-Hispanic White	1298 (33.67%)	303 (33.82%)	
Non-Hispanic Black	810 (21.01%)	188 (20.98%)	
Others	702 (18.21%)	148 (16.52%)	
Parity (%)			<0.01
1–3	1503 (67.13%)	447 (59.68%)	
4–6	637 (28.45%)	252 (33.64%)	
7–9	82 (3.66%)	43 (5.74%)	
> 10	17 (0.76%)	7 (0.93%)	
Hypertension (%)			<0.001
Yes	548 (14.22%)	336 (37.50%)	
No	3305 (85.78%)	560 (62.50%)	
Hypercholesterolemia (%)			<0.001
Yes	511 (13.66%)	360 (40.45%)	
No	3230 (86.34%)	530 (59.55%)	
Diabetes (%)			<0.001
Yes	201 (5.22%)	148 (16.54%)	
No	3651 (94.78%)	747 (83.46%)	
Smoking status (%)			<0.001
Yes	1016 (27.12%)	321 (35.83%)	
No	2730 (72.88%)	575 (64.17%)	
Heavy alcohol (%)			0.003
Yes	221 (7.59%)	74 (11.11%)	
No	2689 (92.41%)	592 (88.89%)	
Vigorous work activity (%)			0.004
Yes	592 (15.36%)	104 (11.62%)	
No	3262 (84.64%)	791 (88.38%)	

**Notes:** Mean ± SD for continuous variables and P value was calculated by linear regression model. % for categorical variables and P value was calculated by chi-square test.

### Associations of fat mass distribution with protein, carbohydrate and fat intake

Our analysis revealed distinct correlations between body fat and the intake of the three major nutrients. As shown in **Table 2**, in the analysis of total participants, after adjusting for age, race, energy intake, parity, hypertension, hypercholesterolemia, diabetes, smoking status, heavy alcohol and vigorous work activity, all the fat indices and protein intake were negatively correlated (TPF: β = -0.017, 95% CI: -0.030, -0.005; APF: β = -0.028, 95% CI: -0.044, -0.012; GPF: β = -0.019, 95% CI: -0.030, -0.008; A/G: β = -0.000, 95% CI: -0.001- -0.000; SAT: β = -1.092, 95% CI: -2.690, 0.506; VAT: β = -0.163, 95% CI: -0.677, 0.351), while they were positively correlated with fat intake (TPF: β = 0.013, 95% CI: -0.001, 0.028; APF: β = 0.014, 95% CI: -0.005, 0.034; GPF: β = 0.007, 95% CI: -0.006, 0.020; A/G: β = 0.000, 95% CI: -0.000–0.000; SAT: β = 2.769, 95% CI: 0.860, 4.678; VAT: β = 0.612, 95% CI: -0.002, 1.226). As for carbohydrate intake, TPF, APF, and GPF were positively correlated (TPF: β = 0.004, 95% CI: -0.001, 0.010; APF: β = 0.002, 95% CI: -0.005, 0.010; GPF: β = 0.007, 95% CI: 0.002, 0.012), while A/G, SAT and VAT were negatively correlated (A/G: β = -0.000, 95% CI: -0.000–0.000; SAT: β = -0.200, 95% CI: -0.904, 0.504; VAT: β = -0.052, 95% CI: -0.278, 0.174), and only the positive correlation with GPT had statistical significance.

**Table 2:** The associations of fat mass distribution with protein, carbohydrate and fat intakes.

Exposures	Outcomes β (95% CI) P value					
	TPF (%)	APF (%)	GPF (%)	A/G	SAT	VAT
**Total**						
Protein intake (g)	-0.017 (-0.030, -0.005) <0.01	-0.028 (-0.044, -0.012) <0.001	-0.019 (-0.030, -0.008) <0.001	-0.000 (-0.001, 0.000) 0.08	-1.092 (-2.690, 0.506) 0.18	-0.163 (-0.677, 0.351) 0.53
Carbohydrate intake (g)	0.004 (-0.001, 0.010) 0.12	0.002 (-0.005, 0.010) 0.50	0.007 (0.002, 0.012) <0.01	-0.000 (-0.000, 0.000) 0.38	-0.200 (-0.904, 0.504) 0.58	-0.052 (-0.278, 0.174) 0.65
Fat intake (g)	0.013 (-0.001, 0.028) 0.07	0.014 (-0.005, 0.034) 0.15	0.007 (-0.006, 0.020) 0.30	0.000 (-0.000, 0.000) 0.58	2.769 (0.860, 4.678) <0.01	0.612 (-0.002, 1.226) 0.05

**Notes:** Age, race, energy intake, parity, hypertension, hypercholesterolemia, diabetes, smoking status, heavy alcohol and vigorous work activity were adjusted.

### Associations of fat mass distribution with protein, carbohydrate and fat intake in pre- and post-menopausal women

**Table 3** spresented the analysis of the relationships between fat mass distribution (including TPF, APF, GPF, A/G, SAT, VAT) and the intake of protein, carbohydrates, and fats across different menopausal statuses (pre-menopausal and post-menopausal).

**Table 3:** Analysis of fat mass distribution with protein, carbohydrate, and fat intake across different menopausal statuses.

** **	**Pre-menopausal**	**Post-menopausal**	**P interaction**
**TPF (%)**			
Protein intake (g)	-0.016 (-0.031, -0.002) 0.0280	0.010 (-0.018, 0.037) 0.4786	0.1
Carbohydrate intake (g)	0.004 (-0.003, 0.010) 0.2495	-0.006 (-0.015, 0.004) 0.2596	0.11
Fat intake (g)	0.027 (0.010, 0.044) 0.0021	0.042 (0.013, 0.072) 0.0051	0.38
**APF (%)**			
Protein intake (g)	-0.030 (-0.049, -0.010) 0.0028	0.026 (-0.011, 0.063) 0.1658	<0.01
Carbohydrate intake (g)	0.003 (-0.006, 0.012) 0.4751	-0.018 (-0.031, -0.005) 0.0070	<0.01
Fat intake (g)	0.027 (0.004, 0.051) 0.0212	0.052 (0.012, 0.092) 0.0116	0.3
**GPF (%)**			
Protein intake (g)	-0.020 (-0.032, -0.008) 0.0015	-0.013 (-0.036, 0.011) 0.2844	0.6
Carbohydrate intake (g)	0.007 (0.001, 0.012) 0.0150	0.004 (-0.005, 0.012) 0.3788	0.53
Fat intake (g)	0.018 (0.004, 0.033) 0.0150	0.025 (0.000, 0.051) 0.0492	0.63
**A/G**			
Protein intake (g)	-0.000 (-0.001, -0.000) 0.0430	0.001 (0.000, 0.002) 0.0054	<0.001
Carbohydrate intake (g)	-0.000 (-0.000, 0.000) 0.8596	-0.001 (-0.001, -0.000) <0.001	<0.001
Fat intake (g)	0.000 (-0.000, 0.001) 0.4965	0.001 (-0.000, 0.001) 0.1561	0.37
**SAT**			
Protein intake (g)	-1.007 (-2.838, 0.823) 0.2809	3.216 (-0.278, 6.709) 0.0713	0.03
Carbohydrate intake (g)	0.042 (-0.778, 0.863) 0.9198	-1.502 (-2.725, -0.279) 0.0162	0.04
Fat intake (g)	3.935 (1.741, 6.129) 0.0004	5.388 (1.622, 9.154) 0.0051	0.51
**VAT**			
Protein intake (g)	-0.069 (-0.676, 0.538) 0.8241	0.728 (-0.430, 1.887) 0.2180	0.23
Carbohydrate intake (g)	-0.036 (-0.308, 0.236) 0.7949	-0.600 (-1.005, -0.195) 0.0037	0.02
Fat intake (g)	0.716 (-0.012, 1.443) 0.0540	2.283 (1.034, 3.532) 0.0003	0.03

**Notes:** Age, race, energy intake, parity, hypertension, hypercholesterolemia, diabetes, smoking status, and vigorous work activity were adjusted.

#### Protein intake

Across various fat mass distribution indices (TPF, APF, A/G, SAT, VAT), except for GPF, the correlations with protein intake switched from negative in pre-menopausal women to positive in post-menopausal women. However, significant interaction effects were observed with APF, A/G, and SAT, indicating a statistically significant shift. That is to say, in pre-menopausal women, protein intake was negatively associated with APF, A/G, and SAT, shifting to a positive association in post-menopausal women. This suggests that menopausal status significantly influences the relationship between protein intake and these three specific fat mass distributions.

#### Carbohydrate intake

Regarding carbohydrate intake, while the table showed varied associations with these fat mass indices, significant interaction effects were notably present with APF, A/G, SAT and VAT. In pre-menopausal women, carbohydrate intake exhibited a non-significant correlation with these four fat distribution indices, but in post-menopausal women, the association turned to be significantly negative. This implies that, carbohydrate intake’s impact on APF, A/G, SAT and VAT is modulated by menopausal status, emphasizing a negative correlation in the post-menopausal phase.

#### Fat intake

The relationship between fat intake and body fat distribution showed that, except for a significant interaction effect on VAT across menopausal statuses, fat intake consistently exhibited a positive correlation with all other fat distribution indices before and after menopause, without significant differences. The positive association between increased fat intake and VAT was more pronounced in post-menopausal women, indicating that while fat intake consistently contributes to visceral fat accumulation, its impact is amplified in post-menopausal women.

**Fig 3:**
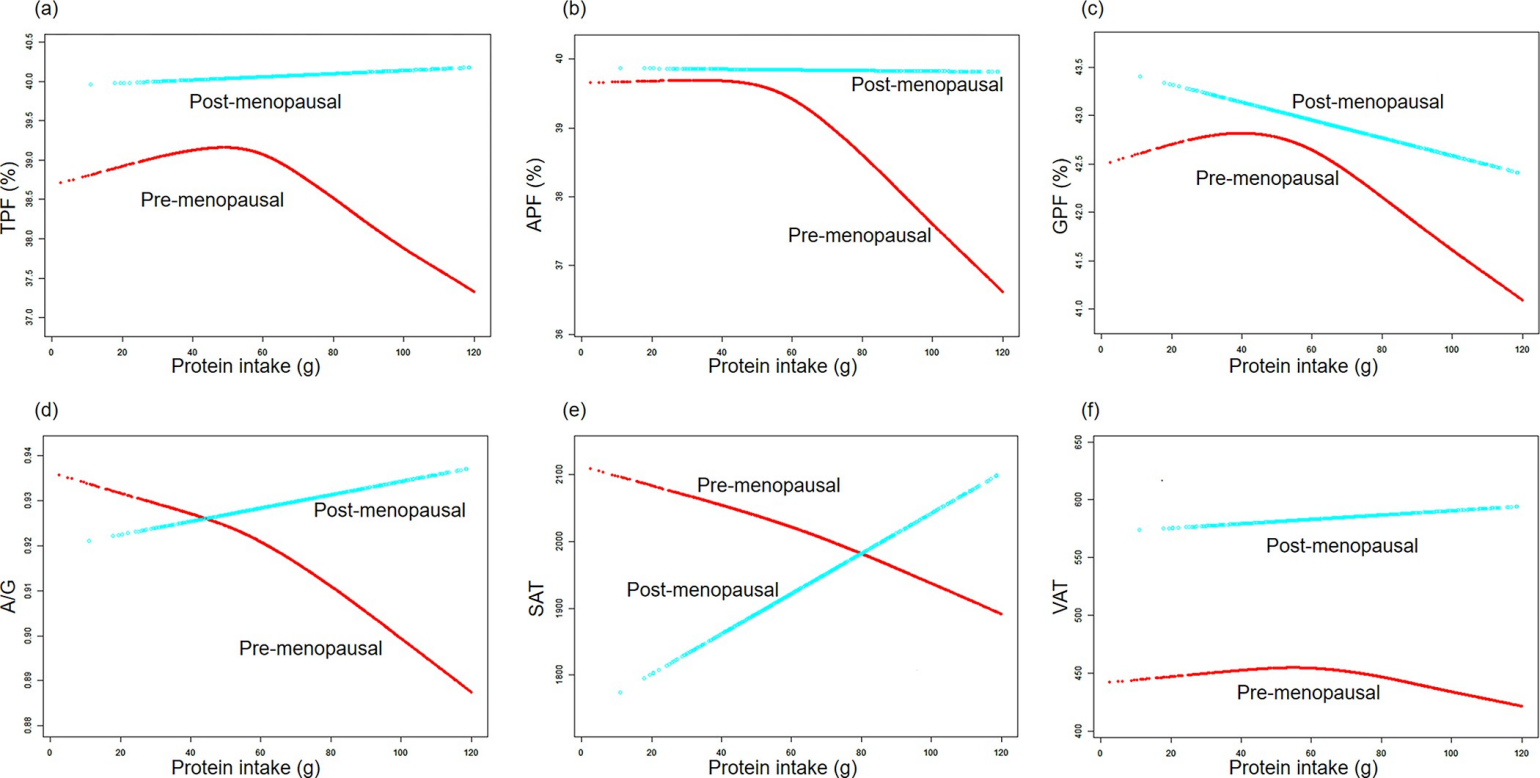
The associations between fat mass distribution and protein intake. **(a)-(f)** Associations of TPF, APF, GPF, A/G, SAT, VAT with protein intake stratified menopausal status. Age, race, energy intake, parity, hypertension, hypercholesterolemia, diabetes, smoking status, heavy alcohol and vigorous work activity were adjusted.

**Fig 4:**
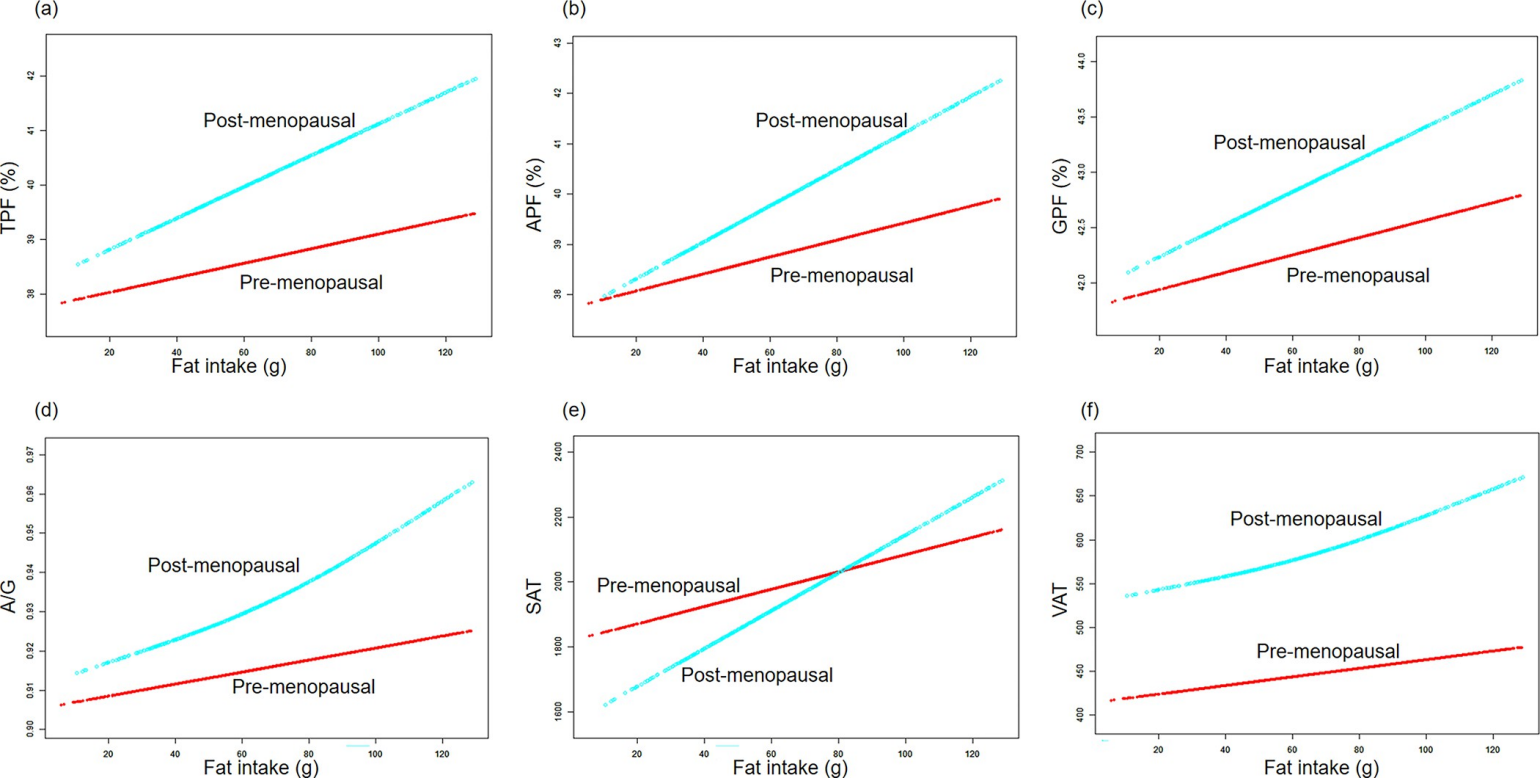
The associations between fat mass distribution and fat intake. **(a)-(f)** Associations of TPF, APF, GPF, A/G, SAT, VAT with fat intake stratified menopausal status. Age, race, energy intake, parity, hypertension, hypercholesterolemia, diabetes, smoking status, heavy alcohol and vigorous work activity were adjusted.

**Fig 5:**
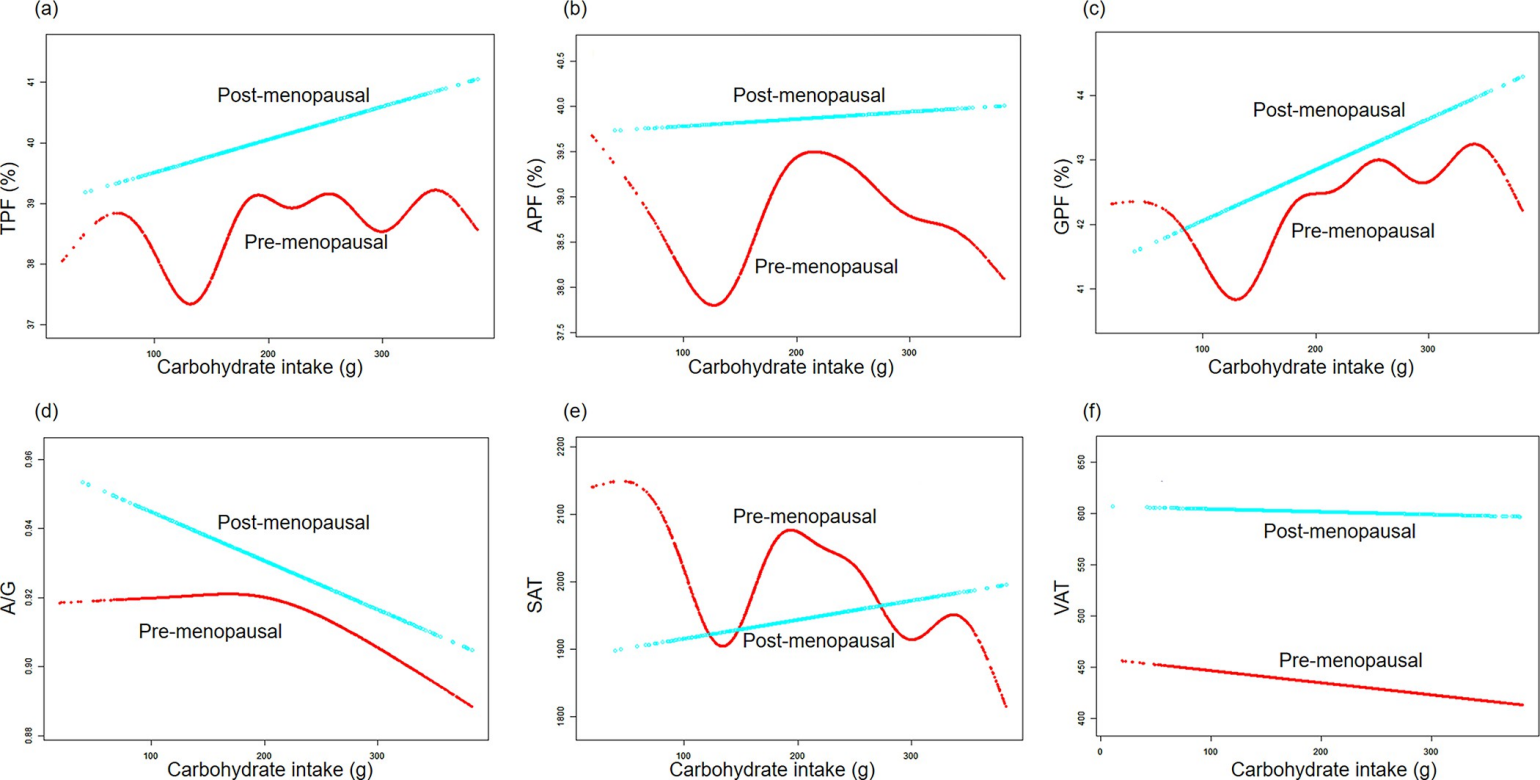
The associations between fat mass distribution and carbohydrate intake. **(a)-(f)** Associations of TPF, APF, GPF, A/G, SAT, VAT with carbohydrate intake stratified menopausal status. Age, race, energy intake, parity, hypertension, hypercholesterolemia, diabetes, smoking status, heavy alcohol and vigorous work activity were adjusted.

Subsequently, we utilized smooth curve fittings to capture the potential non-linear relationships stratified by menopausal status between body fat outcomes and protein, fat, and carbohydrate intake. As depicted in **Fig 3**, pre-menopausal women displayed a decrease in all the fat indices (TPF, APF, GPF, A/G, SAT and VAT) as protein intake increased. In contrast, post-menopausal women exhibited an opposite trend, except for GPF. Furthermore, **Fig 4** demonstrated that both pre- and post-menopausal women showed an increase in all body fat outcomes with higher fat intake. In **Fig 5**, the regression results indicated that only the decrease in A/G and VAT with an increase in carbohydrate intake were captured.

## Discussion

Our study sheds light on the intricate relationships between nutrient intake, body fat distribution, and menopausal status in a diverse population of women based on data from the NHANES. We have observed significant associations between protein, carbohydrate, and fat intake and various measures of body fat distribution, underscoring the importance of dietary factors in shaping adiposity patterns. Importantly, our findings highlight differences in these associations between premenopausal and postmenopausal women. While premenopausal women generally displayed negative correlations between protein intake and body fat distribution, postmenopausal women exhibited more complex patterns, with positive correlations between protein and fat intake and specific fat distribution measures. These results emphasize the need for tailored dietary guidelines that consider women’s unique nutritional requirements at different stages of their reproductive life. As the global prevalence of obesity continues to rise, understanding the interplay between diet, body composition, and health outcomes is crucial for the development of effective strategies for obesity prevention and management. Our study contributes to this understanding and underscores the importance of considering both nutrient intake and menopausal status in future research and public health initiatives aimed at improving women’s health and well-being.

Protein is widely recognized as a crucial macronutrient in weight-loss strategies [34], with various proposed mechanisms including enhanced satiety hormone secretion (glucose-dependent insulinotropic polypeptide (GIP) and glucagon-like peptide-1 (GLP-1)), reduced hunger hormone secretion (ghrelin), increased thermic effect of food, and protein-induced improvements in gluconeogenesis and glucose homeostasis [35, 36]. Moreover, restricting carbohydrate intake has been suggested to enhance fat mass loss through increased fat oxidation in high-protein diets [37]. The relationship between carbohydrates and obesity remains a topic of intense debate, as studies have reported positive associations between certain dietary carbohydrates (refined carbohydrates and sugar) and weight gain, while others focusing on unrefined carbohydrates have suggested opposing effects [38, 39]. As our analysis did not record refined or unrefined carbohydrate intake, prospective studies are needed to elucidate the relationship between obesity and specific carbohydrate types. In contrast, compelling evidence supports the notion that a high-fat diet promotes elevated energy intake and body weight [11]. Animal studies have demonstrated that unrestricted access to high-fat diets leads to increased appetite activity and obesity, with obese rats maintaining similar body weight to low-fat diet-fed counterparts but exhibiting greater fat mass gain [40]. Consistent with our study findings, body fat showed a positive association with fat intake in both pre- and post-menopausal women.

Menopause is accompanied by significant hormonal changes, particularly a decrease in estrogen levels [12, 41]. This hormonal transition leads to various physiological alterations, including decreased bone mass density, subcutaneous fat redistribution to the visceral area, insulin resistance, increased cardiovascular disease risk, and diminished quality of life [42]. Consistent with previous research, our study revealed higher body fat indices and lower nutrient intakes in post-menopausal women. For example, Jubrias et al. reported that post-menopausal women exhibited double the amount of non-contractile muscle tissue, such as intramuscular fat, compared to younger women [43]. What’s more, our analysis demonstrated different trends of body fat with protein intake among pre- and post-menopausal women. The decline in estrogen levels may be associated with decreased food consumption, insulin resistance, inflammation, macrophage recruitment, reduced energy expenditure, and impaired mitochondrial function [44], potentially impacting the role of protein in fat loss. Additionally, hormones such as testosterone and dehydroepiandrosterone (DHEA) may play a certain role. While testosterone supplementation in men has been linked to increased muscle mass [45], post-menopausal women experience decreased bioavailable testosterone levels [46]. Similarly, DHEA has been associated with increased muscle mass and decreased fat mass [47], but its circulating levels decline with age, particularly during menopause [48]. Nevertheless, further investigations are warranted to elucidate the direct mechanisms underlying the influence of menopause on body fat and protein intake.

This study possesses several notable strengths. Firstly, it was a large-scale investigation involving a nationally representative sample of American women, allowing for comprehensive assessment of fat mass distribution and dietary intake. Secondly, to our knowledge, it was the first study to investigate the relationship between fat mass distribution and intake of the three major nutrients, stratified by menopausal status. Thirdly, the measurement of adipose tissue in different body regions was conducted using DXA, which is recognized for its accuracy in assessing adiposity.

However, despite these strengths, there are several limitations that should be acknowledged. Firstly, the cross-sectional design of the study restricts our ability to establish causal relationships. Secondly, the exclusion of participants without DXA scans may introduce bias in the body fat mass data. Thirdly, the analysis stratified by menopausal status resulted in smaller sample sizes within each category, potentially leading to non-significant results in some of the multivariable regression analyses. Another limitation of our study is that the reliance on data solely pertaining to macronutrient intake and vigorous physical activity, which does not directly capture the comprehensive energy balance of the participants. The utilization of macronutrients for energy metabolism is influenced by the individual’s energy balance status. While protein and carbohydrate oxidation can be adjusted to achieve balance with their intake, fat intake is primarily influenced by the gap between energy intake (from protein and carbohydrates) and energy expenditure, rather than its direct impact on oxidation. Consequently, future prospective studies with larger sample sizes are warranted to further explore these associations.

## Conclusion

In conclusion, our study reveals significant associations between nutrient intake and body fat distribution among women, with notable differences observed between premenopausal and postmenopausal individuals. These findings underscore the importance of tailored dietary recommendations that account for women’s changing nutritional needs throughout their reproductive life. This research contributes valuable insights into the complex relationship between diet, body composition, and health outcomes, offering potential avenues for more effective strategies in the prevention and management of obesity in women.
